# Supplementary material for: DOC concentrations across a depth-dependent light gradient on a Caribbean coral reef
Source: PeerJ. 2017 Jun 12;5:e3456. doi: 10.7717/peerj.3456 (PMC5470576; doi:10.7717/peerj.3456)
Supplement: Supplemental Information 1 [file peerj-05-3456-s001.docx]

**Randomized distances along 30 m transect lines (5, 10, and 20 m depth) alongside which photo quadrats (1 x 1 m) were placed alternatingly on both sides to determine benthic composition.** Two photo quadrats of the 5 m transect and five of the 20 m transect had to be excluded from be analysis due to insufficient quality (marked with an asterisk).

| **Randomized distances along the transects** | | | |
| --- | --- | --- | --- |
| **side** | **5 m** | **10 m** | **20 m** |
| right | 0.36 | 0.46 | 0.78 |
| left | 1.12 | 1.12 | *1.41 |
| right | 2.13 | 2.22 | 2.96 |
| left | 3.41 | 4.33 | 4.63 |
| right | 4.52 | 5.46 | 5.56 |
| left | 5.65 | 9.33 | *8.38 |
| right | *6.01 | 10.75 | 9.39 |
| left | 7.26 | 12.63 | 11.37 |
| right | 8.04 | 14.20 | 14.36 |
| left | 9.63 | 17.18 | 15.14 |
| right | 11.00 | 18.14 | 17.05 |
| left | *13.42 | 19.44 | 18.36 |
| right | 19.87 | 20.36 | *19.53 |
| left | 22.39 | 21.75 | *20.72 |
| right | 23.75 | 22.57 | 22.25 |
| left | 25.59 | 23.21 | 23.76 |
| right | 26.77 | 24.37 | 25.11 |
| left | 27.27 | 26.51 | *26.99 |
| right | 28.07 | 27.90 | 28.33 |
| left | 29.77 | 28.47 | 29.90 |

**Benthic composition determined from photo quadrats (1 x 1 m) placed at randomized distances and alternatingly on both sides along a 30 m transect line (see above).** Percentage cover of most dominant benthic components was quantified from randomly-generated overlaid points on each photograph using Coral Point Count with Excel Extensions (CPCe).

| **SUBCATEGORIES (% of transect)** |  |  |  |  | **Continue SPONGE (SP)** | **5 m** | **10 m** | **20 m** |
| --- | --- | --- | --- | --- | --- | --- | --- | --- |
| **MACROALGAE (MA)** | **5 m** | **10 m** | **20 m** |  | Aplysina archeri (APAR) | 7.0 | 0.0 | 0.0 |
| Dictyota (DIC) | 0.1 | 8.9 | 0.1 |  | Aplysina cauliformis (APCA) | 0.0 | 0.0 | 0.0 |
| Halimeda (HAL) | 0.0 | 0.1 | 0.0 |  | Aplysina fistularis (APFI) | 0.0 | 0.0 | 0.0 |
| Lobophora (LOB) | 0.0 | 1.4 | 0.0 |  | Aplysina lacunosa (APLA) | 0.0 | 0.0 | 0.0 |
| Sargassum (SAR) | 0.0 | 0.0 | 0.0 |  | Callyspongia fallax (CAFA) | 0.0 | 0.0 | 0.0 |
| Ulva (ULV) | 0.0 | 0.0 | 0.0 |  | Callyspongia plicifera (CAPL) | 0.0 | 0.0 | 0.0 |
| other algae (OAL) | 0.1 | 3.4 | 0.1 |  | Callyspongia vaginalis (CAVA) | 0.0 | 0.0 | 0.0 |
| other algae (OA) | 0.0 | 0.3 | 0.0 |  | Chondrilla caribensis (CHCA) | 0.0 | 0.0 | 0.0 |
| **TURF ALGAE (TA)** |  |  |  |  | Clathria curacaoensis (CLCU) | 0.0 | 0.0 | 0.0 |
| turf algae (TA) | 0.0 | 9.3 | 0.0 |  | Desmapsamma anchorata (DEAN) | 0.0 | 0.0 | 0.0 |
| **CRUSTOSE CORALLINE ALGAE (CCA)** | |  |  |  | Ectyoplasia ferox (ECFE) | 0.0 | 0.0 | 0.0 |
| crustose coralline algae (CCA) | 0.0 | 3.5 | 0.0 |  | Erylus formosus (ERFO) | 0.0 | 0.0 | 0.0 |
| **BENTHIC CYANOBACTERIAL MAT (BCM)** | |  |  |  | Haliclona vansoesti (HAVA) | 0.0 | 0.0 | 0.0 |
| Lymbia (LYM) | 0.0 | 0.0 | 0.0 |  | Halisarca caerulea (HACA) | 0.0 | 0.0 | 0.0 |
| brown cyanobacterial mat (BRO) | 0.0 | 6.1 | 0.0 |  | Ircinia campana (IRCA) | 0.0 | 0.3 | 0.0 |
| other cyanobateria (OCY) | 0.0 | 0.0 | 0.0 |  | Ircinia felix (IRFE) | 0.0 | 0.0 | 0.0 |
| red cyanobacterial mat (RED) | 0.0 | 0.5 | 0.0 |  | Ircinia strobolina (IRST) | 0.0 | 0.1 | 0.0 |
| shade cyanobacteria (SHA) | 0.0 | 0.0 | 0.0 |  | Monanchora arbuscula (MOAR) | 0.0 | 0.0 | 0.0 |
| **HARD CORAL (HC)** |  |  |  |  | Mycale laxissima (MYLA) | 0.0 | 0.0 | 0.0 |
| Argaricia (ARG) | 0.0 | 2.5 | 0.0 |  | Neofibularia nolitangere (NENO) | 0.0 | 0.3 | 0.0 |
| Colpophyllia (COL) | 0.0 | 0.5 | 0.0 |  | Niphates erecta (NIER) | 0.0 | 0.0 | 0.0 |
| Dendrogyra (DEN) | 0.0 | 0.0 | 0.0 |  | Petrosia weinbergi (PEWE) | 0.0 | 0.0 | 0.0 |
| Diploria (DIP) | 0.0 | 0.3 | 0.0 |  | Plakortis angulospiculatus (PLAN) | 0.0 | 0.0 | 0.0 |
| Madracis mirabilis (MM) | 0.0 | 16.9 | 0.0 |  | Plakortis halichondrioides (PLHA) | 0.0 | 0.0 | 0.0 |
| Montastraea annularis (MA) | 1.3 | 0.4 | 1.3 |  | Scopalina ruetzleri (SCRU) | 0.0 | 0.0 | 0.0 |
| Montastraea cavernosa (MC) | 0.0 | 0.3 | 0.0 |  | Spirastrella hartmani (SPHA) | 0.0 | 0.0 | 0.0 |
| Montastraea faveolata (MF) | 0.4 | 2.6 | 0.4 |  | Verongula rigida (VERI) | 0.0 | 0.0 | 0.0 |
| Porites (POR) | 0.3 | 0.3 | 0.3 |  | Xestospongia muta (XESP) | 0.0 | 0.0 | 0.0 |
| Siderastrea (SID) | 0.0 | 0.0 | 0.0 |  | other sponge (OS) | 0.0 | 0.8 | 0.3 |
| other coral (OC) | 0.0 | 0.8 | 0.0 |  | **TUNICATE (TU)** |  |  |  |
| **MILLEPORA (ML)** |  |  |  |  | tunicate (TU) | 0.0 | 0.1 | 0.0 |
| Millepora (ML) | 0.0 | 0.6 | 0.0 |  | **SAND (SA)** |  |  |  |
| **GORGONIAN (GG)** |  |  |  |  | sand (SA) | 0.0 | 23.1 | 73.5 |
| gorgonian (GG) | 0.0 | 0.3 | 0.0 |  | **CORAL ROCK (RCK)** |  |  |  |
| **SPONGE (SP)** |  |  |  |  | coral rock (RCK) | 0.0 | 4.3 | 11.0 |
| Agelas clathrodes (AGCL) | 0.0 | 0.0 | 0.0 |  | **CORAL RUBBLE (CR)** |  |  |  |
| Agelas conifera (AGCO) | 0.0 | 0.1 | 0.0 |  | coral rubble (CR) | 0.0 | 11.1 | 13.1 |
| Agelas dispar (AGDI) | 0.0 | 0.0 | 0.0 |  | **OTHER (O)** |  |  |  |
| Aiolochroia crassa (AICR) | 0.0 | 0.0 | 0.0 |  | other (O) | 0.0 | 1.3 | 0.0 |

**Sampling and analysis of bacterial samples**

Bacterial concentrations in the water column 2 m off the reef slope (towards the open ocean) was sampled using a modified bilge pump connected to a low density polyethylene collapsible jerry can (19 L, Cole-Parmer) (for details see Dinsdale et al. 2008). To avoid potential contamination jerry cans and the bilge pump were washed once with 10% bleach and three times with fresh water before the dive. In between the individual samplings, the bilge pumps were additionally flushed with reef water. In June, 2015 bacterial samples (n = 5) were taken in the study area at 15 m depth. Water samples were transported to the nearby CARMABI research station and one subsample (9 mL) was taken from each jerry can within 1 h. Subsamples were fixed in 4% paraformaldehyde and filtered over a 0.2 μm polycarbonate filter (Millipore, 25 mm) and a 0.45 μm HA support filter (Millipore, 25 mm). Filters were air-dried and stored in plastic tubes at -20˚C until analysis at the CARMABI research station. After mounting on a microscopy slide in a DAPI-mix, bacteria were counted on an epifluorescence microscope (1250 x). Per filter 10 grids (36 x36 μm, divided into 10 rows and columns) were counted or up to a minimum of 200 bacteria.

**Bacterial concentrations (n=5) taken at Snake Bay, at 15 m depth, in the water column, 2 m off the reef slope.**

| **#** | **Bacterial concentration [cells L^-1^]** | |
| --- | --- | --- |
| **1** | 9.0 x 10^5^ |  |
| **2** | 8.1 x 10^5^ | **Mean:** |
| **3** | 1.1 x 10^6^ | 9.6 x 10^5^ |
| **4** | 9.9 x 10^5^ | **SD:** |
| **5** | 9.9 x 10^5^ | 1.2 x 10^5^ |

***In situ* DOC concentrations (n=5) measured in the water column (2 m off the reef slope; water) and at the substrate-water interfaces of the reef algae *Dictyota* sp. (algae) and the scleractinain coral *Orbicella faveolata* (coral) at 5, 10 and 20 m depth.** Concentrations marked with an asterisk were excluded from the analysis due to irregularities during the injection and measurement procedure of the samples.

|  |  | **DOC concentration [μmol C L^-1^]** | | | | |
| --- | --- | --- | --- | --- | --- | --- |
| **Type** | **Depth** | **1** | **2** | **3** | **4** | **5** |
| **water** | 5 m | 102 | 104 | 85 | 84 | 78 |
|  | 10 m | 90 | 92 | 89 | 98 | *122 |
|  | 20 m | 81 | 80 | 92 | 97 | 88 |
| **algae** | 5 m | *162 | 82 | 85 | 95 | 88 |
|  | 10 m | 107 | 100 | 90 | 108 | 112 |
|  | 20 m | 81 | 90 | 84 | 80 | 77 |
| **coral** | 5 m | 85 | 88 | 93 | 93 | 86 |
|  | 10 m | 86 | 98 | 81 | 83 | 103 |
|  | 20 m | 99 | 88 | 94 | 90 | 85 |

**Ambient light intensity (PAR) measured during the water sampling at 20, 10, and 5 m depth.**

| **Depth** | **Time** | **Light intensity**  **[μmol photons m^-2^ s^-1^]** |  | **Depth** | **Time** | **Light intensity**  **[μmol photons m^-2^ s^-1^]** |
| --- | --- | --- | --- | --- | --- | --- |
| 20 m | 12:13 | 328 |  | 10 m | 12:34 | 615 |
| 20 m | 12:14 | 193 |  | 10 m | 12:35 | 762 |
| 20 m | 12:15 | 409 |  | 10 m | 12:36 | 715 |
| 20 m | 12:16 | 396 |  | 10 m | 12:37 | 695 |
| 20 m | 12:17 | 373 |  | 5 m | 12:38 | 1132 |
| 20 m | 12:18 | 447 |  | 5 m | 12:39 | 1107 |
| 20 m | 12:19 | 393 |  | 5 m | 12:40 | 1718 |
| 20 m | 12:20 | 367 |  | 5 m | 12:41 | 1087 |
| 20 m | 12:21 | 407 |  | 5 m | 12:42 | 978 |
| 20 m | 12:22 | 348 |  | 5 m | 12:43 | 1122 |
| 20 m | 12:23 | 456 |  | 5 m | 12:44 | 1599 |
| 10 m | 12:26 | 736 |  | 5 m | 12:45 | 1328 |
| 10 m | 12:27 | 865 |  | 5 m | 12:46 | 840 |
| 10 m | 12:28 | 772 |  | 5 m | 12:47 | 1186 |
| 10 m | 12:29 | 578 |  | 5 m | 12:48 | 1145 |
| 10 m | 12:30 | 725 |  | 5 m | 12:49 | 851 |
| 10 m | 12:31 | 672 |  | 5 m | 12:50 | 1176 |
| 10 m | 12:32 | 615 |  | 5 m | 12:51 | 1726 |
| 10 m | 12:33 | 678 |  |  |  |  |

**Light intensity (PAR) and temperature (˚C) recorded on March 17, 2017 during the *ex situ* experiment to determine maximum excitation pressure over photosystem II in the reef alga *Dictyota* sp.** Light intensity is depicted in blue, temperature in orange. For raw data see table below.

**
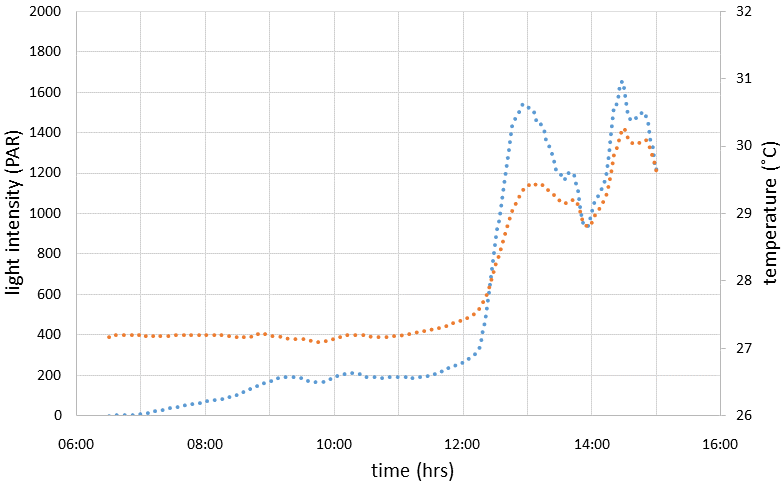
**

| **time** | **PAR** | **˚C** |  | **time** | **PAR** | **˚C** |  | **time** | **PAR** | **˚C** |  | **time** | **PAR** | **˚C** |
| --- | --- | --- | --- | --- | --- | --- | --- | --- | --- | --- | --- | --- | --- | --- |
| 06:00:00 | 0 | 27.1 |  | 08:16:00 | 99 | 27.1 |  | 10:32:00 | 239 | 27.2 |  | 12:48:00 | 1630 | 29.6 |
| 06:01:00 | 0 | 27.1 |  | 08:17:00 | 101 | 27.1 |  | 10:33:00 | 209 | 27.2 |  | 12:49:00 | 1593 | 29.6 |
| 06:02:00 | 0 | 27.2 |  | 08:18:00 | 106 | 27.2 |  | 10:34:00 | 199 | 27.2 |  | 12:50:00 | 1591 | 29.6 |
| 06:03:00 | 0 | 27.1 |  | 08:19:00 | 111 | 27.2 |  | 10:35:00 | 198 | 27.2 |  | 12:51:00 | 1678 | 29.7 |
| 06:04:00 | 0 | 27.1 |  | 08:20:00 | 125 | 27.2 |  | 10:36:00 | 194 | 27.2 |  | 12:52:00 | 1686 | 29.7 |
| 06:05:00 | 1 | 27.1 |  | 08:21:00 | 133 | 27.1 |  | 10:37:00 | 193 | 27.2 |  | 12:53:00 | 1722 | 29.8 |
| 06:06:00 | 0 | 27.3 |  | 08:22:00 | 130 | 27.2 |  | 10:38:00 | 189 | 27.2 |  | 12:54:00 | 1764 | 29.8 |
| 06:07:00 | 1 | 27.2 |  | 08:23:00 | 129 | 27.2 |  | 10:39:00 | 187 | 27.2 |  | 12:55:00 | 1637 | 29.8 |
| 06:08:00 | 0 | 27.2 |  | 08:24:00 | 123 | 27.3 |  | 10:40:00 | 189 | 27.2 |  | 12:56:00 | 1444 | 29.7 |
| 06:09:00 | 1 | 27.3 |  | 08:25:00 | 120 | 27.1 |  | 10:41:00 | 187 | 27.2 |  | 12:57:00 | 1467 | 29.7 |
| 06:10:00 | 0 | 27.1 |  | 08:26:00 | 123 | 27.3 |  | 10:42:00 | 191 | 27.2 |  | 12:58:00 | 1337 | 29.5 |
| 06:11:00 | 0 | 27.3 |  | 08:27:00 | 145 | 27.1 |  | 10:43:00 | 191 | 27.2 |  | 12:59:00 | 1319 | 29.5 |
| 06:12:00 | 0 | 27.1 |  | 08:28:00 | 150 | 27.3 |  | 10:44:00 | 189 | 27.2 |  | 13:00:00 | 1307 | 29.4 |
| 06:13:00 | 0 | 27.2 |  | 08:29:00 | 147 | 27.3 |  | 10:45:00 | 190 | 27.2 |  | 13:01:00 | 1331 | 29.4 |
| 06:14:00 | 0 | 27.3 |  | 08:30:00 | 145 | 27.2 |  | 10:46:00 | 192 | 27.2 |  | 13:02:00 | 1315 | 29.4 |
| 06:15:00 | 0 | 27.2 |  | 08:31:00 | 139 | 27.2 |  | 10:47:00 | 184 | 27.2 |  | 13:03:00 | 1328 | 29.3 |
| 06:16:00 | 0 | 27.2 |  | 08:32:00 | 147 | 27.2 |  | 10:48:00 | 185 | 27.2 |  | 13:04:00 | 1450 | 29.4 |
| 06:17:00 | 0 | 27.2 |  | 08:33:00 | 154 | 27.2 |  | 10:49:00 | 185 | 27.3 |  | 13:05:00 | 1225 | 29.4 |
| 06:18:00 | 0 | 27.2 |  | 08:34:00 | 163 | 27.2 |  | 10:50:00 | 189 | 27.2 |  | 13:06:00 | 655 | 29.0 |
| 06:19:00 | 0 | 27.2 |  | 08:35:00 | 149 | 27.2 |  | 10:51:00 | 193 | 27.2 |  | 13:07:00 | 1011 | 28.9 |
| 06:20:00 | 0 | 27.2 |  | 08:36:00 | 151 | 27.2 |  | 10:52:00 | 193 | 27.3 |  | 13:08:00 | 1429 | 29.2 |
| 06:21:00 | 0 | 27.2 |  | 08:37:00 | 153 | 27.2 |  | 10:53:00 | 187 | 27.3 |  | 13:09:00 | 1509 | 29.3 |
| 06:22:00 | 0 | 27.1 |  | 08:38:00 | 171 | 27.2 |  | 10:54:00 | 185 | 27.2 |  | 13:10:00 | 1325 | 29.4 |
| 06:23:00 | 0 | 27.1 |  | 08:39:00 | 183 | 27.5 |  | 10:55:00 | 183 | 27.2 |  | 13:11:00 | 1221 | 29.4 |
| 06:24:00 | 0 | 27.3 |  | 08:40:00 | 168 | 27.3 |  | 10:56:00 | 179 | 27.2 |  | 13:12:00 | 1094 | 29.2 |
| 06:25:00 | 0 | 27.2 |  | 08:41:00 | 164 | 27.1 |  | 10:57:00 | 177 | 27.2 |  | 13:13:00 | 1217 | 29.3 |
| 06:26:00 | 0 | 27.3 |  | 08:42:00 | 157 | 27.1 |  | 10:58:00 | 169 | 27.2 |  | 13:14:00 | 1316 | 29.3 |
| 06:27:00 | 0 | 27.2 |  | 08:43:00 | 171 | 27.2 |  | 10:59:00 | 174 | 27.2 |  | 13:15:00 | 960 | 29.1 |
| 06:28:00 | 0 | 27.1 |  | 08:44:00 | 183 | 27.2 |  | 11:00:00 | 188 | 27.2 |  | 13:16:00 | 638 | 28.8 |
| 06:29:00 | 0 | 27.2 |  | 08:45:00 | 153 | 27.8 |  | 11:01:00 | 203 | 27.3 |  | 13:17:00 | 1354 | 29.1 |
| 06:30:00 | 1 | 27.3 |  | 08:46:00 | 180 | 27.5 |  | 11:02:00 | 209 | 27.3 |  | 13:18:00 | 1401 | 29.2 |
| 06:31:00 | 1 | 27.1 |  | 08:47:00 | 168 | 27.3 |  | 11:03:00 | 206 | 27.3 |  | 13:19:00 | 1318 | 29.3 |
| 06:32:00 | 1 | 27.3 |  | 08:48:00 | 165 | 27.2 |  | 11:04:00 | 184 | 27.3 |  | 13:20:00 | 1271 | 29.3 |
| 06:33:00 | 1 | 27.8 |  | 08:49:00 | 177 | 27.2 |  | 11:05:00 | 188 | 27.3 |  | 13:21:00 | 1235 | 29.4 |
| 06:34:00 | 1 | 27.2 |  | 08:50:00 | 178 | 27.2 |  | 11:06:00 | 189 | 27.3 |  | 13:22:00 | 1187 | 29.3 |
| 06:35:00 | 1 | 27.2 |  | 08:51:00 | 177 | 27.2 |  | 11:07:00 | 190 | 27.3 |  | 13:23:00 | 1346 | 29.4 |
| 06:36:00 | 1 | 27.2 |  | 08:52:00 | 177 | 27.1 |  | 11:08:00 | 190 | 27.3 |  | 13:24:00 | 1151 | 29.3 |
| 06:37:00 | 2 | 27.2 |  | 08:53:00 | 173 | 27.1 |  | 11:09:00 | 189 | 27.3 |  | 13:25:00 | 696 | 28.9 |
| 06:38:00 | 1 | 27.1 |  | 08:54:00 | 173 | 27.1 |  | 11:10:00 | 186 | 27.3 |  | 13:26:00 | 1053 | 29.0 |
| 06:39:00 | 2 | 27.2 |  | 08:55:00 | 177 | 27.1 |  | 11:11:00 | 183 | 27.3 |  | 13:27:00 | 1193 | 29.1 |
| 06:40:00 | 2 | 27.1 |  | 08:56:00 | 194 | 27.1 |  | 11:12:00 | 179 | 27.3 |  | 13:28:00 | 1305 | 29.3 |
| 06:41:00 | 3 | 27.3 |  | 08:57:00 | 195 | 27.1 |  | 11:13:00 | 179 | 27.3 |  | 13:29:00 | 1216 | 29.3 |
| 06:42:00 | 3 | 27.2 |  | 08:58:00 | 189 | 27.1 |  | 11:14:00 | 189 | 27.3 |  | 13:30:00 | 932 | 29.1 |
| 06:43:00 | 3 | 27.1 |  | 08:59:00 | 168 | 27.1 |  | 11:15:00 | 191 | 27.3 |  | 13:31:00 | 821 | 28.9 |
| 06:44:00 | 4 | 27.4 |  | 09:00:00 | 185 | 27.1 |  | 11:16:00 | 191 | 27.3 |  | 13:32:00 | 1317 | 29.1 |
| 06:45:00 | 5 | 27.2 |  | 09:01:00 | 219 | 27.1 |  | 11:17:00 | 203 | 27.3 |  | 13:33:00 | 1406 | 29.3 |
| **time** | **PAR** | **˚C** |  | **time** | **PAR** | **˚C** |  | **time** | **PAR** | **˚C** |  | **time** | **PAR** | **˚C** |
| 06:46:00 | 5 | 27.3 |  | 09:02:00 | 222 | 27.2 |  | 11:18:00 | 208 | 27.3 |  | 13:34:00 | 1309 | 29.4 |
| 06:47:00 | 7 | 27.1 |  | 09:03:00 | 226 | 27.2 |  | 11:19:00 | 213 | 27.3 |  | 13:35:00 | 1241 | 29.4 |
| 06:48:00 | 8 | 27.2 |  | 09:04:00 | 229 | 27.2 |  | 11:20:00 | 215 | 27.3 |  | 13:36:00 | 1272 | 29.4 |
| 06:49:00 | 9 | 27.3 |  | 09:05:00 | 215 | 27.2 |  | 11:21:00 | 216 | 27.3 |  | 13:37:00 | 1333 | 29.5 |
| 06:50:00 | 10 | 27.2 |  | 09:06:00 | 205 | 27.2 |  | 11:22:00 | 204 | 27.3 |  | 13:38:00 | 1341 | 29.5 |
| 06:51:00 | 12 | 27.2 |  | 09:07:00 | 193 | 27.2 |  | 11:23:00 | 224 | 27.3 |  | 13:39:00 | 1289 | 29.5 |
| 06:52:00 | 13 | 27.3 |  | 09:08:00 | 189 | 27.2 |  | 11:24:00 | 227 | 27.3 |  | 13:40:00 | 1193 | 29.4 |
| 06:53:00 | 15 | 27.3 |  | 09:09:00 | 195 | 27.2 |  | 11:25:00 | 227 | 27.3 |  | 13:41:00 | 1208 | 29.4 |
| 06:54:00 | 16 | 27.2 |  | 09:10:00 | 203 | 27.2 |  | 11:26:00 | 221 | 27.3 |  | 13:42:00 | 1193 | 29.4 |
| 06:55:00 | 17 | 27.3 |  | 09:11:00 | 209 | 27.2 |  | 11:27:00 | 224 | 27.3 |  | 13:43:00 | 754 | 29.0 |
| 06:56:00 | 19 | 27.2 |  | 09:12:00 | 205 | 27.2 |  | 11:28:00 | 229 | 27.4 |  | 13:44:00 | 401 | 28.6 |
| 06:57:00 | 21 | 27.1 |  | 09:13:00 | 189 | 27.2 |  | 11:29:00 | 233 | 27.4 |  | 13:45:00 | 305 | 28.3 |
| 06:58:00 | 21 | 27.2 |  | 09:14:00 | 178 | 27.2 |  | 11:30:00 | 235 | 27.4 |  | 13:46:00 | 258 | 28.0 |
| 06:59:00 | 23 | 27.1 |  | 09:15:00 | 176 | 27.2 |  | 11:31:00 | 235 | 27.4 |  | 13:47:00 | 231 | 27.8 |
| 07:00:00 | 24 | 27.1 |  | 09:16:00 | 178 | 27.2 |  | 11:32:00 | 241 | 27.4 |  | 13:48:00 | 251 | 27.7 |
| 07:01:00 | 25 | 27.2 |  | 09:17:00 | 180 | 27.1 |  | 11:33:00 | 237 | 27.4 |  | 13:49:00 | 320 | 27.7 |
| 07:02:00 | 27 | 27.2 |  | 09:18:00 | 180 | 27.1 |  | 11:34:00 | 241 | 27.4 |  | 13:50:00 | 359 | 27.7 |
| 07:03:00 | 28 | 27.2 |  | 09:19:00 | 167 | 27.1 |  | 11:35:00 | 243 | 27.4 |  | 13:51:00 | 660 | 28.0 |
| 07:04:00 | 29 | 27.2 |  | 09:20:00 | 181 | 27.2 |  | 11:36:00 | 247 | 27.4 |  | 13:52:00 | 1045 | 28.4 |
| 07:05:00 | 31 | 27.2 |  | 09:21:00 | 180 | 27.2 |  | 11:37:00 | 247 | 27.4 |  | 13:53:00 | 1387 | 28.9 |
| 07:06:00 | 31 | 27.1 |  | 09:22:00 | 165 | 27.1 |  | 11:38:00 | 251 | 27.4 |  | 13:54:00 | 655 | 28.6 |
| 07:07:00 | 31 | 27.3 |  | 09:23:00 | 157 | 27.1 |  | 11:39:00 | 254 | 27.4 |  | 13:55:00 | 915 | 28.7 |
| 07:08:00 | 31 | 27.2 |  | 09:24:00 | 155 | 27.1 |  | 11:40:00 | 256 | 27.4 |  | 13:56:00 | 1875 | 29.4 |
| 07:09:00 | 32 | 27.2 |  | 09:25:00 | 162 | 27.1 |  | 11:41:00 | 256 | 27.4 |  | 13:57:00 | 1413 | 29.5 |
| 07:10:00 | 35 | 27.1 |  | 09:26:00 | 168 | 27.1 |  | 11:42:00 | 257 | 27.4 |  | 13:58:00 | 1897 | 29.9 |
| 07:11:00 | 35 | 27.3 |  | 09:27:00 | 152 | 27.1 |  | 11:43:00 | 257 | 27.4 |  | 13:59:00 | 1821 | 30.1 |
| 07:12:00 | 36 | 27.2 |  | 09:28:00 | 147 | 27.1 |  | 11:44:00 | 258 | 27.5 |  | 14:00:00 | 1590 | 30.1 |
| 07:13:00 | 37 | 27.2 |  | 09:29:00 | 151 | 27.1 |  | 11:45:00 | 261 | 27.5 |  | 14:01:00 | 1327 | 30.0 |
| 07:14:00 | 39 | 27.2 |  | 09:30:00 | 155 | 27.1 |  | 11:46:00 | 267 | 27.5 |  | 14:02:00 | 1466 | 30.0 |
| 07:15:00 | 39 | 27.2 |  | 09:31:00 | 144 | 27.1 |  | 11:47:00 | 269 | 27.5 |  | 14:03:00 | 1478 | 29.9 |
| 07:16:00 | 41 | 27.2 |  | 09:32:00 | 143 | 27.1 |  | 11:48:00 | 267 | 27.5 |  | 14:04:00 | 1559 | 30.0 |
| 07:17:00 | 41 | 27.2 |  | 09:33:00 | 146 | 27.1 |  | 11:49:00 | 268 | 27.5 |  | 14:05:00 | 1753 | 30.2 |
| 07:18:00 | 43 | 27.2 |  | 09:34:00 | 144 | 27.1 |  | 11:50:00 | 271 | 27.5 |  | 14:06:00 | 1761 | 30.3 |
| 07:19:00 | 43 | 27.3 |  | 09:35:00 | 150 | 27.1 |  | 11:51:00 | 267 | 27.5 |  | 14:07:00 | 1768 | 30.4 |
| 07:20:00 | 45 | 27.3 |  | 09:36:00 | 162 | 27.1 |  | 11:52:00 | 269 | 27.5 |  | 14:08:00 | 1691 | 30.4 |
| 07:21:00 | 47 | 27.3 |  | 09:37:00 | 189 | 27.1 |  | 11:53:00 | 271 | 27.5 |  | 14:09:00 | 1629 | 30.4 |
| 07:22:00 | 49 | 27.2 |  | 09:38:00 | 196 | 27.1 |  | 11:54:00 | 281 | 27.5 |  | 14:10:00 | 1606 | 30.3 |
| 07:23:00 | 50 | 27.2 |  | 09:39:00 | 179 | 27.1 |  | 11:55:00 | 289 | 27.5 |  | 14:11:00 | 1659 | 30.4 |
| 07:24:00 | 51 | 27.4 |  | 09:40:00 | 173 | 27.1 |  | 11:56:00 | 286 | 27.5 |  | 14:12:00 | 1765 | 30.5 |
| 07:25:00 | 53 | 27.2 |  | 09:41:00 | 173 | 27.1 |  | 11:57:00 | 291 | 27.5 |  | 14:13:00 | 1720 | 30.5 |
| 07:26:00 | 53 | 27.1 |  | 09:42:00 | 177 | 27.1 |  | 11:58:00 | 299 | 27.5 |  | 14:14:00 | 1550 | 30.4 |
| 07:27:00 | 54 | 27.3 |  | 09:43:00 | 187 | 27.2 |  | 11:59:00 | 305 | 27.5 |  | 14:15:00 | 1471 | 30.3 |
| 07:28:00 | 53 | 27.2 |  | 09:44:00 | 189 | 27.2 |  | 12:00:00 | 321 | 27.5 |  | 14:16:00 | 1765 | 30.4 |
| 07:29:00 | 54 | 27.2 |  | 09:45:00 | 188 | 27.2 |  | 12:01:00 | 334 | 27.6 |  | 14:17:00 | 1737 | 30.4 |
| 07:30:00 | 55 | 27.1 |  | 09:46:00 | 183 | 27.2 |  | 12:02:00 | 348 | 27.6 |  | 14:18:00 | 1702 | 30.4 |
| 07:31:00 | 57 | 27.3 |  | 09:47:00 | 165 | 27.2 |  | 12:03:00 | 357 | 27.6 |  | 14:19:00 | 1795 | 30.5 |
| **time** | **PAR** | **˚C** |  | **time** | **PAR** | **˚C** |  | **time** | **PAR** | **˚C** |  | **time** | **PAR** | **˚C** |
| 07:32:00 | 57 | 27.2 |  | 09:48:00 | 167 | 27.2 |  | 12:04:00 | 359 | 27.6 |  | 14:20:00 | 1529 | 30.4 |
| 07:33:00 | 57 | 27.2 |  | 09:49:00 | 187 | 27.2 |  | 12:05:00 | 363 | 27.6 |  | 14:21:00 | 655 | 29.6 |
| 07:34:00 | 59 | 27.2 |  | 09:50:00 | 221 | 27.2 |  | 12:06:00 | 374 | 27.7 |  | 14:22:00 | 1638 | 30.0 |
| 07:35:00 | 59 | 27.2 |  | 09:51:00 | 215 | 27.2 |  | 12:07:00 | 379 | 27.7 |  | 14:23:00 | 1813 | 30.2 |
| 07:36:00 | 61 | 27.2 |  | 09:52:00 | 231 | 27.2 |  | 12:08:00 | 387 | 27.7 |  | 14:24:00 | 1854 | 30.5 |
| 07:37:00 | 62 | 27.2 |  | 09:53:00 | 238 | 27.2 |  | 12:09:00 | 381 | 27.8 |  | 14:25:00 | 1866 | 30.6 |
| 07:38:00 | 63 | 27.3 |  | 09:54:00 | 230 | 27.2 |  | 12:10:00 | 394 | 27.8 |  | 14:26:00 | 1904 | 30.7 |
| 07:39:00 | 63 | 27.2 |  | 09:55:00 | 210 | 27.2 |  | 12:11:00 | 402 | 27.8 |  | 14:27:00 | 1947 | 30.9 |
| 07:40:00 | 63 | 27.2 |  | 09:56:00 | 231 | 27.2 |  | 12:12:00 | 415 | 27.9 |  | 14:28:00 | 1615 | 30.7 |
| 07:41:00 | 65 | 27.1 |  | 09:57:00 | 261 | 27.3 |  | 12:13:00 | 425 | 27.9 |  | 14:29:00 | 971 | 30.0 |
| 07:42:00 | 66 | 27.1 |  | 09:58:00 | 247 | 27.3 |  | 12:14:00 | 457 | 28.0 |  | 14:30:00 | 521 | 29.4 |
| 07:43:00 | 67 | 27.3 |  | 09:59:00 | 231 | 27.3 |  | 12:15:00 | 655 | 28.1 |  | 14:31:00 | 408 | 28.9 |
| 07:44:00 | 68 | 27.3 |  | 10:00:00 | 203 | 27.3 |  | 12:16:00 | 910 | 28.2 |  | 14:32:00 | 393 | 28.6 |
| 07:45:00 | 69 | 27.2 |  | 10:01:00 | 223 | 27.3 |  | 12:17:00 | 1272 | 28.4 |  | 14:33:00 | 387 | 28.4 |
| 07:46:00 | 70 | 27.1 |  | 10:02:00 | 229 | 27.3 |  | 12:18:00 | 1281 | 28.6 |  | 14:34:00 | 1119 | 28.8 |
| 07:47:00 | 71 | 27.3 |  | 10:03:00 | 213 | 27.3 |  | 12:19:00 | 1314 | 28.7 |  | 14:35:00 | 1861 | 29.6 |
| 07:48:00 | 73 | 27.2 |  | 10:04:00 | 206 | 27.3 |  | 12:20:00 | 1363 | 28.8 |  | 14:36:00 | 1823 | 30.0 |
| 07:49:00 | 73 | 27.2 |  | 10:05:00 | 200 | 27.2 |  | 12:21:00 | 1367 | 28.8 |  | 14:37:00 | 1862 | 30.3 |
| 07:50:00 | 75 | 27.3 |  | 10:06:00 | 177 | 27.2 |  | 12:22:00 | 1353 | 28.8 |  | 14:38:00 | 1887 | 30.5 |
| 07:51:00 | 76 | 27.3 |  | 10:07:00 | 177 | 27.2 |  | 12:23:00 | 1412 | 28.9 |  | 14:39:00 | 1453 | 30.2 |
| 07:52:00 | 77 | 27.2 |  | 10:08:00 | 181 | 27.2 |  | 12:24:00 | 1459 | 29.0 |  | 14:40:00 | 1483 | 30.3 |
| 07:53:00 | 77 | 27.2 |  | 10:09:00 | 190 | 27.2 |  | 12:25:00 | 1467 | 29.0 |  | 14:41:00 | 1809 | 30.5 |
| 07:54:00 | 79 | 27.3 |  | 10:10:00 | 197 | 27.2 |  | 12:26:00 | 1477 | 29.1 |  | 14:42:00 | 1873 | 30.6 |
| 07:55:00 | 79 | 27.3 |  | 10:11:00 | 221 | 27.2 |  | 12:27:00 | 1479 | 29.1 |  | 14:43:00 | 1836 | 30.7 |
| 07:56:00 | 80 | 27.2 |  | 10:12:00 | 239 | 27.2 |  | 12:28:00 | 1462 | 29.1 |  | 14:44:00 | 1761 | 30.7 |
| 07:57:00 | 81 | 27.2 |  | 10:13:00 | 225 | 27.2 |  | 12:29:00 | 1494 | 29.2 |  | 14:45:00 | 1694 | 30.6 |
| 07:58:00 | 81 | 27.2 |  | 10:14:00 | 205 | 27.2 |  | 12:30:00 | 1471 | 29.2 |  | 14:46:00 | 1761 | 30.6 |
| 07:59:00 | 80 | 27.3 |  | 10:15:00 | 178 | 27.2 |  | 12:31:00 | 1471 | 29.2 |  | 14:47:00 | 1733 | 30.7 |
| 08:00:00 | 83 | 27.1 |  | 10:16:00 | 158 | 27.2 |  | 12:32:00 | 1426 | 29.2 |  | 14:48:00 | 1805 | 30.7 |
| 08:01:00 | 83 | 27.2 |  | 10:17:00 | 157 | 27.2 |  | 12:33:00 | 1404 | 29.2 |  | 14:49:00 | 1117 | 30.2 |
| 08:02:00 | 84 | 27.2 |  | 10:18:00 | 161 | 27.2 |  | 12:34:00 | 1490 | 29.2 |  | 14:50:00 | 995 | 29.8 |
| 08:03:00 | 85 | 27.2 |  | 10:19:00 | 165 | 27.1 |  | 12:35:00 | 1509 | 29.3 |  | 14:51:00 | 425 | 29.2 |
| 08:04:00 | 85 | 27.3 |  | 10:20:00 | 181 | 27.2 |  | 12:36:00 | 1447 | 29.3 |  | 14:52:00 | 428 | 28.8 |
| 08:05:00 | 85 | 27.3 |  | 10:21:00 | 215 | 27.2 |  | 12:37:00 | 1492 | 29.3 |  | 14:53:00 | 456 | 28.5 |
| 08:06:00 | 86 | 27.2 |  | 10:22:00 | 215 | 27.2 |  | 12:38:00 | 1443 | 29.3 |  | 14:54:00 | 1392 | 29.1 |
| 08:07:00 | 87 | 27.2 |  | 10:23:00 | 201 | 27.2 |  | 12:39:00 | 1422 | 29.3 |  | 14:55:00 | 1578 | 29.5 |
| 08:08:00 | 87 | 27.2 |  | 10:24:00 | 187 | 27.2 |  | 12:40:00 | 1453 | 29.3 |  | 14:56:00 | 1363 | 29.6 |
| 08:09:00 | 89 | 27.3 |  | 10:25:00 | 175 | 27.2 |  | 12:41:00 | 1431 | 29.3 |  | 14:57:00 | 400 | 29.0 |
| 08:10:00 | 86 | 27.2 |  | 10:26:00 | 174 | 27.2 |  | 12:42:00 | 1505 | 29.3 |  | 14:58:00 | 367 | 28.6 |
| 08:11:00 | 89 | 27.2 |  | 10:27:00 | 169 | 27.2 |  | 12:43:00 | 1597 | 29.4 |  | 14:59:00 | 486 | 28.4 |
| 08:12:00 | 93 | 27.3 |  | 10:28:00 | 149 | 27.2 |  | 12:44:00 | 1622 | 29.5 |  | 15:00:00 | 939 | 28.5 |
| 08:13:00 | 95 | 27.1 |  | 10:29:00 | 154 | 27.2 |  | 12:45:00 | 1631 | 29.5 |  |  |  |  |
| 08:14:00 | 94 | 27.1 |  | 10:30:00 | 177 | 27.2 |  | 12:46:00 | 1604 | 29.6 |  |  |  |  |
| 08:15:00 | 97 | 27.2 |  | 10:31:00 | 229 | 27.2 |  | 12:47:00 | 1605 | 29.6 |  |  |  |  |

**Maximum potential quantum yield (F*v*/F*m*) and effective quantum yield; (ΔF/F*m*’) measured in *Dictyota* sp. to assess maximum excitation pressure over photosystem II.**

| **maximum potential quantum yield (Fv/Fm)** | | | |  | **effective quantum yieldΔF/Fm’)** | | | |
| --- | --- | --- | --- | --- | --- | --- | --- | --- |
| **yield** | **#** | **mean** | **sd** |  | **yield** | **#** | **mean** | **sd** |
| 0.636 | 1 | 0.67 | 0.05 |  | 0.548 | 1 | 0.49 | 0.05 |
| 0.649 |  |  |  |  | 0.478 |  | 0.51 |  |
| 0.736 |  |  |  |  | 0.441 |  |  |  |
| 0.768 | 2 | 0.73 | 0.03 |  | 0.498 | 2 | 0.54 | 0.11 |
| 0.729 |  |  |  |  | 0.465 |  | 0.56 |  |
| 0.705 |  |  |  |  | 0.671 |  |  |  |
| 0.673 | 3 | 0.62 | 0.06 |  | 0.609 | 3 | 0.52 | 0.09 |
| 0.559 |  |  |  |  | 0.441 |  |  |  |
| 0.626 |  |  |  |  | 0.5 |  |  |  |
| 0.656 | 4 | 0.64 | 0.02 |  | 0.359 | 4 | 0.46 | 0.11 |
| 0.64 |  |  |  |  | 0.43 |  |  |  |
| 0.616 |  |  |  |  | 0.581 |  |  |  |
| 0.649 | 5 | 0.66 | 0.01 |  | 0.279 | 5 | 0.25 | 0.04 |
| 0.661 |  |  |  |  | 0.277 |  |  |  |
| 0.658 |  |  |  |  | 0.206 |  |  |  |
| 0.597 | 6 | 0.62 | 0.05 |  | 0.276 | 6 | 0.35 | 0.12 |
| 0.591 |  |  |  |  | 0.491 |  |  |  |
| 0.68 |  |  |  |  | 0.292 |  |  |  |
| 0.719 | 7 | 0.71 | 0.01 |  | 0.393 | 7 | 0.41 | 0.02 |
| 0.694 |  |  |  |  | 0.392 |  |  |  |
| 0.702 |  |  |  |  | 0.435 |  |  |  |
| 0.618 | 8 | 0.62 | 0.01 |  | 0.344 | 8 | 0.45 | 0.10 |
| 0.613 |  |  |  |  | 0.438 |  |  |  |
| 0.636 |  |  |  |  | 0.553 |  |  |  |
| 0.67 | 9 | 0.65 | 0.02 |  | 0.61 | 9 | 0.52 | 0.12 |
| 0.642 |  |  |  |  | 0.376 |  |  |  |
| 0.645 |  |  |  |  | 0.56 |  |  |  |
| 0.643 | 10 | 0.60 | 0.11 |  | 0.358 | 10 | 0.37 | 0.05 |
| 0.679 |  |  |  |  | 0.327 |  |  |  |
| 0.467 |  |  |  |  | 0.429 |  |  |  |
